# Determination of the characteristic inactivation fluence for SARS-CoV-2 under UV-C radiation considering light absorption in culture media

**DOI:** 10.1038/s41598-021-94648-w

**Published:** 2021-07-27

**Authors:** Juan Carlos Martínez-Antón, Alejandro Brun, Daniel Vázquez, Sandra Moreno, Antonio A. Fernández-Balbuena, Javier Alda

**Affiliations:** 1 Applied Optics Complutense Group, Faculty of Optics and Optometry, University Complutense of Madrid, Av. Arcos de Jalón, 118, 28037 Madrid, Spain; 2 Centro de Investigación en Sanidad Animal, Instituto Nacional de Investigación y Tecnología Agraria y Alimentaria, Carretera Algete-El Casar de Talamanca, Km 8.1, 28130 Valdeolmos, Madrid Spain

**Keywords:** Biophysics, Applied optics

## Abstract

The optical absorption coefficient of culture media is critical for the survival analysis of pathogens under optical irradiation. The quality of the results obtained from experiments relies on the optical analysis of the spatial distribution of fluence which also depends on the geometry of the sample. In this contribution, we consider both the geometrical shape and the culture medium’s absorption coefficient to evaluate how the spatial distribution of optical radiation affects pathogens/viruses. In this work, we exposed SARS-CoV-2 to UV-C radiation ($\lambda$ = 254 nm) and we calculated—considering the influence of the optical absorption of the culture medium—a characteristic inactivation fluence of $F_i$ = 4.7 J/m^2^, or an equivalent 10% survival (D90 dose) of 10.8 J/m^2^. Experimentally, we diluted the virus into sessile drops of Dulbecco’s Modified Eagle Medium to evaluate pathogen activity after controlled doses of UV irradiation. To validate the optical absorption mode, we carried out an additional experiment where we varied droplet size. Our model—including optical absorption and geometrical considerations—provides robust results among a variety of experimental situations, and represents our experimental conditions more accurately. These results will help to evaluate the capability of UV disinfecting strategies applied to a variety of everyday situations, including the case of micro-droplets generated by respiratory functions.

## Introduction

The covid19 pandemic has ignited a worldwide interest in mitigating the effects of the virus: a variety of methods have been proposed to inactivate the causative pathogen. Current techniques are based on biochemical inactivation through virucidal substances^1–4^, on exposure to high temperatures^5,6^, and on light irradiation at virucidal wavelengths^7–15^, among others. Light’s germicidal effect is relevant in the spectral range between 100 and 280 nm, which is also known as the UV-C band. Several light sources in this range have demonstrated the capability to inactivate the virus and many other pathogens (e.g. *Escherichia coli*, *Salmonella typhimurium*, *Acanthamoeba castellanii*, etc. )^13,15–17^.

Fluence, *F*, is the key variable in the pathogen UV inactivation process. It is defined as the optical energy per area unit, J/m^2^, and can be calculated as the product of irradiance, in W/m^2^, and time, in s. We consider an exponential decay of the ratio, $\eta$, between the number of active viruses, $N_s$, after being irradiated with a fluence *F*, and the number of active viruses before irradiation, $N_0$, to obtain the survival ratio as^7^:

1 $$\begin{aligned} \eta = \frac{N_s}{N_0}= \exp ( - F /F_i ) ,\end{aligned}$$

where $F_i$ is the characteristic fluence that expresses the fluence needed to reduce pathogen population to a level of $\eta = 1/e=0.3679$, or the D63 associated fluence, $F_i= F_{\mathrm{D}63}$^18^. Therefore, for UV-C light disinfection to be feasible, the characteristic inactivation fluence, $F_i$, must be known as accurately as possible^18–20^. The inverse of this fluence is also known as the susceptibility of the virus, $k=1/F_i$. This approach is specifically valid for single-strand-RNA viruses as SARS-CoV-2^21^. Once the value of $F_i$ is known, it can be easily transformed in to the $F_{\mathrm{D}50}$, $F_{\mathrm{D}90}$, and $F_{\mathrm{D}99}$ fluences for survival ratios of 0.5, 0.1, and 0.01, respectively. The first step to validate UV-C disinfection strategies is to determine $F_i$.

Most measurements that test inactivation techniques require virus exposure in a controlled environment. This procedure typically involves the use of liquids where the pathogen is diluted. A very common culture medium is Dulbecco’s Modified Eagle Medium (DMEM) supplemented with fetal bovine serum. Often, these liquids are mostly opaque to UV radiation: the optical absorption coefficient $\alpha$ is non-negligible. Therefore, fluence diminishes with the Lambert-Beer relation as light travels through the liquid:

2 $$\begin{aligned} F(z) = T F(z=0) \exp ( - \alpha z ) , \end{aligned}$$

where *z* is the distance propagated within the liquid ($z=0$ at the location of the air/liquid interface), and *T* is the transmission coefficient of the air/liquid interface. For normal incidence

3 $$\begin{aligned} T=4n/(n+1)^2 , \end{aligned}$$

where *n* is the index of refraction of the liquid. For the geometry used in this contribution, although the angle of incidence can be larger than 0, the value of *T* does not depart significantly from the normal incidence case. In Eq. (2), $F(z=0)$ is the incident fluence. As *z* increases, the radiation absorbed by the culture medium reduces fluence for virus inactivation. We consider the characteristic fluence, $F_i$, as intrinsic for a particular pathogen, independent of optical attenuation of the surrounding media. In real experiments, we must account for the geometry, photometric setup, and absorption properties of culture medium to extract this parameter correctly.

In literature, we find a variety of set-ups to measure UV susceptibility of pathogens^7–9,22^. A common practice is the use of well plates filled with culture medium where the pathogens are suspended^23,24^. If the absorption at UV is neglected, this approach may be inappropriate. In fact, absorption, or scattering, can be a relevant cause of some of the discrepancies found among different works, even working with the same pathogen^7–9,25^. Moreover, typical well plates are opaque to UV sterilizing radiation: their walls may cast shadows and leave pathogens unexposed. A common solution is to use a collimated light source. In practice, a LED or a masked source just upside each well plate would be enough. However, liquid menisci may also generate refraction shadows with similar consequences even in these controlled situations^26^. Low survival ratios $\eta$ (or equivalent high fluences) can indicate the presence of hidden pathogens. This results in a large survival ratio or a saturation floor for $\eta$ for high fluences, also known as the tail of the inactivation curve^3,21^. Morowitz^27^ proposed a method that considers optical absorption in a well plate. A practical solution is to stir the fluid containing the pathogens to homogenize concentration and radiation exposure^28^. Unfortunately, stirring is not practical for moderate and small samples or fluid volumes.

During the covid19 health crisis, different studies have demonstrated that an important transmission vector is the presence of contaminated micro-droplets in air^29–31^, and on several types of surfaces^5,32,33^. These pathogenic micro-droplets are generated by sneezing, coughing, talking, or even regular respiratory function in humans who are infected by the virus, independently if they develop symptoms requiring medical treatment or not^34–36^. Whether the pathogens are airborne or lie on fomites, the knowledge of the characeristic fluence or susceptibility allows to optimally design sterilizing systems^7^. Experimental setups to analyze the UV susceptibility of airborne pathogens—like those generating measles, influenza and the covid19 itself—are relatively cumbersome and risky^37–39^. We believe that our method will improve the calculation of the net UV susceptibility without the difficulties of handling aerosolized pathogens.

In this contribution, we propose a simplified setup: we irradiate small culture media drops deposited on a substrate (plastic or glass) and we apply a specific absorption model, assuming that the drop has a spherical cap shape. All possible shadows are avoided and no stirring is required. Although our experiments are conducted with SARS-CoV-2, our analysis should be valid for any other pathogen susceptible to be cultured or suspended within in a fluid.

This paper is organized as follows. "Materials and methods" section presents our measurement set-up and a model to describe the inactivation process considering absorption. SARS-CoV-2 is exposed to UV-light and the measurements are fitted with our model; we further explain this in "Results and discussion" section. We have focused on comparing two geometries to develop a robust theory and a realistic explanation of the phenomena observed. Finally, we have summarized the main contributions of this paper in "Conclusions".

## Materials and methods

### Irradiation chamber

The number of available sources within the UV-C spectrum is quite limited. Some works propose and use excimer lamps showing virucidal action at at $\lambda =222$ nm (exposure claimed to be low risk for humans)^15^. Moreover, there is an increased interest in using UV-LED at the UV-C band because its compactness and its foreseeable improvement in efficiency^13,21^. In this analysis, we have used low-pressure Hg lamps because, so far, their efficiency in the line emission at $\lambda _{\mathrm{Hg}}=253.7$ nm is remarkably higher when compared with other options (excimer lamps, UV-LEDs, etc.).

The lamps have a custom-made enclosure that, after characterization, were placed in the biosafety lab. We incorporated an additional set of UV radiometers inside the chamber to register the actual irradiance at the sample’s location. To comply with safety standards, we have also monitored the ozone level generated from the lamps with ozone-meters (Gasman-03-A Crowcom Detection Instruments): our sources are not an ozone generation hazard. The inactivation box contained two lamps Osram Germicidal HNS G5 6W generating 1.7W at the UV-C emission line. These light sources were placed 36 cm above the irradiated plane, generating an irradiance of $\sim$14 W/m^2^. Most of the experiments were carried out with a reduced UV-C output. This decrease in irradiance was accomplished by masking the lamp with a narrow window of approximately 5 mm wide. Under these conditions, our 254 nm UV-C light sources produced an irradiance of $\sim 0.7$ W/m^2^, with values ranging between 0.65 and 0.80 W/m^2^ across the plane where the samples were placed. The actual values of irradiance were measured every time the exposure was done, and its variability across the plane of interest was taken into account. The inactivation box was radiometrically characterized with a RMD UVC Opsytec radiometer. This equipment was calibrated on May the 5th, 2020 by Opsytec Dr. Gröbel GmbH. During the calibration of the irradiation chamber, we also established a 6 minutes warm-up time for stable irradiance levels during measurements. The inactivation box included a mechanical shutter that shielded the volume for the samples from the UV radiation, and allowed a safe operation of the system. Before every UV irradiation, the inactivation camera was prepared by first allowing the lamps to stabilize, and by registering the actual values of the irradiance at the plane of interest.

### Virus sample preparation and UV irradiation

We used the SARS-related coronavirus 2 strain NL/2020/ (BetaCoV/Netherlands/01). It was received from European Virus Archive GLOBAL (EVA-GLOBAL) and kindly provided by Dr. Richard Molenkamp from the Erasmus University Medical Center (Rotterdam). For all the experiments presented here, a virus stock was generated by infecting Vero E6 cells (ATCC-CRL/1586) at low infection multiplicity (0.01 PFU/cell). After a 72 hour period (post-infection), the supernatants were collected, placed into sealed buckets, and clarified by centrifugation for 10 min at 2.000 rpm in an refrigerated Eppendorf 5810 centrifuge. The virus stock was stored at $-80\,^\circ$C for preservation.

Vero E6 cells were routinely maintained in Dulbecco’s modified Eagle’s medium (DMEM) (Biowest) containing supplements (5% fetal bovine serum, 2 mM/mL-glutamine, 100 U/mL penicillin, 100 $\upmu$g/mL streptomycin). The cell flasks were routinely incubated at 37 $^\circ$C in a CO$_2$ atmosphere with 5% humidity.

The titer of the SARS-CoV-2 stock was determined in Vero-E6 cells by plaque assay. Samples were subjected to 3 or 10-fold serial dilutions and added to each well. After 1 h of inoculum adsorption at 37$^\circ$C, the cells were washed with medium and a semisolid mixture of 1% Carboxy-methyl cellulose (CMC) in serum supplemented DMEM added to each well. After the 3 days post-infection period, the wells were examined for the presence of virus induced lysis plaques. The cells were fixed overnight with 10% formaldehyde solution, the semisolid medium was removed and the fixed cultures were further stained for 10 minutes with 2% crystal violet filtered solution. After washing out the staining solution, plaques were visually inspected, and counted in those dilutions displaying (whenever possible) more than 30 lysis plaques. The titer was estimated by the following formula: (number of plaques $\times$ sample dilution factor)/sample volume (in mL).

**Figure 1:**
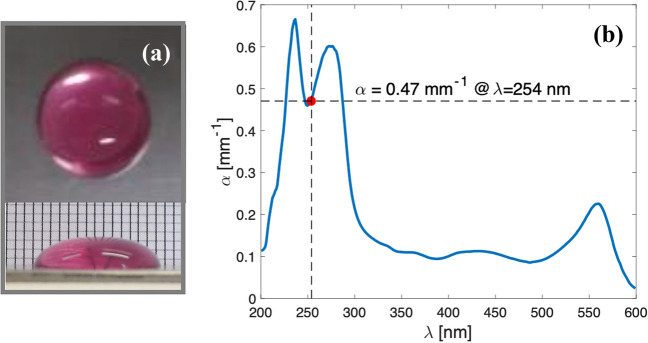
(**a**) Upper and lateral views of one of the DMEM drops used in the preliminary essays of this study. (**b**) Spectral variation of the absorption coefficient, $\alpha$. The red circle represents the value of $\alpha =0.47$ mm$^{-1}$ at the wavelength of emission of the Hg lamps, $\lambda _{\mathrm{Hg}}=254$ nm.

Short-wave ultraviolet light (UV-C) treatment was applied directly on 300 $\upmu$L virus containing culture medium drops (approximate 14–17 mm diameter and 2–3 mm high) in borosilicate cell culture treated slides (Labtek NY) or plastic treated petri dishes (Corning), as shown in Fig. 1a. After exposure to the UV-C light source, drops were collected and immediately frozen for later analysis by plaque forming assay.

All SARS-CoV-2 live culture procedures were conducted in an enhanced biosafety level 3 laboratory (BSL3+). All personnel wore powered air-purifying respirators (3M) incorporated into Prochem suits. Manipulation of live infectious virus was completed inside a biosafety class-II cabinet.

### Model of UV-C propagation through absorbing media

The culture solution described in Virus sample preparation and UV irradiation section is optically absorbing in the UV. We obtained the absorption coefficient of the DMEM by measuring the internal transmittance $T_{\mathrm{int}}$ and by applying the Lambert-Beer law (Eq. (2)) to obtain

4 $$\begin{aligned} \alpha =-\ln {T_{\mathrm{int}}}/d ,\end{aligned}$$

where *d* is the propagation distance within the medium. We used UV-quartz cuvette cells of a thickness of $d=5$ mm. The internal transmittance is

5 $$\begin{aligned} T_{\mathrm{in}t}=I/I_0, \end{aligned}$$

where *I* corresponds to the transmission of a cuvette filled with DMEM, and $I_0$ is the reference transmission to another cuvette filled with pure water. In these measurements, we used a fiber optics UV-Vis spectrophotometer (Avantes AvaSpec-1024-USB2-RM) and a deuterium light source (Avantes AvaLight-D(H)-s). The calculated spectral absorption coefficient is shown in Fig. 1b ($\alpha =0.47 \pm 0.02$ mm$^{-1}$ for $\lambda =254$ nm). Assuming an index of refraction of $n=1.35$, we estimated the transmittance of the air-fluid interface to be larger than 0.97 for a wide angular range of incidence up to 45$^\circ$. This high transmittance enables us to neglect its effect on the experimental results.

**Figure 2:**
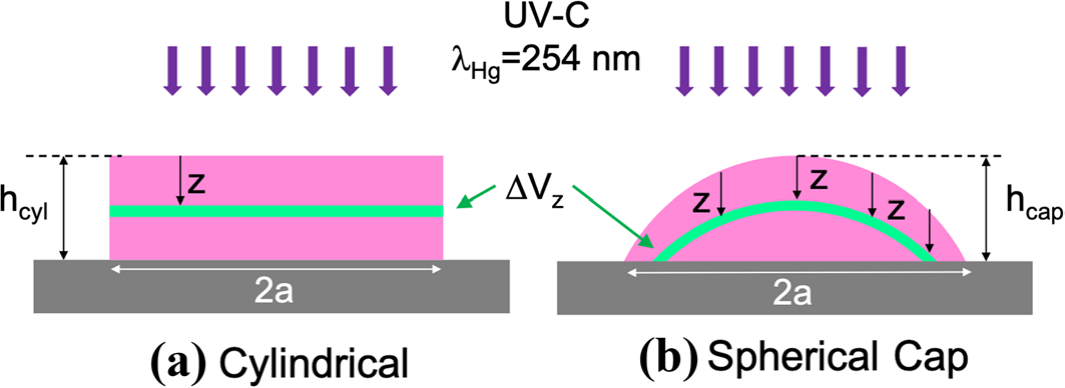
Geometry of the virus culture for two cases: cylindrical (**a**) and spherical cap (**b**). The diameter of the sample is given by 2*a* and its height $h_{{\mathrm{cyl}}}$ and $h_{{\mathrm{cap}}}$ for the cylinder and the spherical cap, respectively.

When an UV-C collimated light propagates through an absorbing material, the irradiance decreases exponentially following the Lambert-Beer equation (see Eq. (2))^7,8,10^. This irradiance dependence also translates into a fluence spatial distribution within the sample. We rely on Eq. (1) when we consider the survival probability as a function of the fluence. By combining these two exponential dependencies (Eqs. (1) and (2)), we can define a survival population for an infinitesimal thin layer, with volume $\Delta V(z)$, situated at a distance *z* from the air/liquid interface as:

6 $$\begin{aligned} \Delta N_s(z) = N_0 \frac{\Delta V(z)}{V_{\mathrm{total}} } \exp \left[ - \frac{1}{F_i} F T \exp ( - \alpha z ) \right] , \end{aligned}$$

where we assume that the total number of viruses before irradiation, $N_0$, is homogeneously distributed within the sample. In this case, the number of virus within the layer is proportional to the volume of the layer, $\Delta V(z)$, as

7 $$\begin{aligned} \Delta N_0(z)= N_0 \Delta V(z)/V_{\mathrm{total}}, \end{aligned}$$

where $V_{\mathrm{total}}$ is the total volume of the sample. From the previous equation, we can clearly see that survival depends on the location of the virus within the volume of the sample through the variable *z*. For cylindrical geometries (see Fig. 2a), the volume of the sample is $V_{\mathrm{total}}= h_{\mathrm{cyl}} A$, where *A* is the area of the transversal section of the cylinder, which can be circular or rectangular, depending on the experiment^7,24^. In a cylinder, every layer has the same volume, and $\Delta N_0(z)$ is constant within the sample. Some authors assume a constant irradiation for all the exposed media, neglecting the effect of optical absorption^11,17^. This last approach could be valid for very shallow drops where the propagation within the absorbing media is negligible. The calculation of the survival ratio for this cylindrical geometry requires the cumulative addition of the survival ratios of every layer within the sample. Mathematically it can be expressed as:

8 $$\begin{aligned} \eta _{\mathrm{cyl}} (F) = \int _0^{h_{\mathrm{cyl}}} \frac{1}{h_{\mathrm{cyl}}} \exp \left[ - \frac{F T}{F_i} \exp ( - \alpha z ) \right] dz , \end{aligned}$$

where $h_{{\mathrm{cyl}}}$ is the total height of the sample (see Fig. 2a). This equation can be seen as the result of the cylindrical geometry model where absorption is considered.

The previous cylindrical model can be refined by further approximations to sample’s shape. If we consider them as drops deposited on a flat surface: the culture media have a spherical cap shape (see Fig. 2b) where the volume of this shape is determined by

9 $$\begin{aligned} V_{\mathrm{cap}} = \frac{\pi h_{\mathrm{cap}}^2}{3} ( 3r-h_{\mathrm{cap}}) , \end{aligned}$$

where $h_{{\mathrm{cap}}}$ is the height of the cap – the height of the droplet – and *r* is the radius of curvature of the spherical air/liquid interface. These two parameters are linked with the diameter of the drop at the bottom flat interface of the substrate, 2*a*. This relation is

10 $$\begin{aligned} 2rh_{\mathrm{cap}}=h_{\mathrm{cap}}^2+ a^2 . \end{aligned}$$

**Figure 3:**
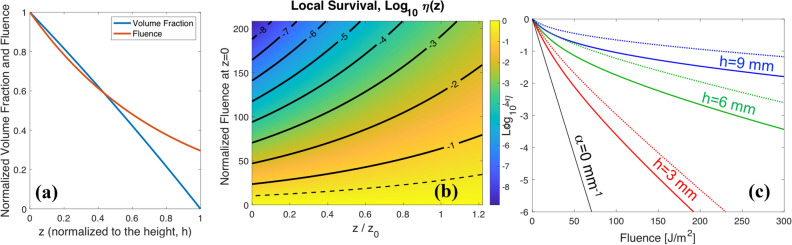
(**a**) Dependence of the volume fraction (in blue), and fluence (in red) when moving from the air/liquid interface, $z=0$, towards the bottom of the drop, $z=h_{\mathrm{cap}}$. In this plot we assumed a value of $h_{\mathrm{cap}}=2.6$ mm, a volume $V=300 \mu$L, and an absorption coefficient $\alpha =0.47$ mm$^{-1}$. (**b**) Local survival rate, $\eta$, in $\log _{10}$ representation for each layer of the drop as a function of the depth within the drop and the incident fluence, *F*. The location within the drop, *z*, is normalized to $z_0=1/\alpha$ where the fluence drops to 1/*e* of the incident fluence. The fluence value is normalized to the characteristic inactivation fluence, $F_i$. The dashed line corresponds to a local survival ratio of 0.37 (related with the characteristics fluence $F_i$ trough Eq. (1)), and the solid lines with labels -1, -2, -3, etc. represent local survival ratios of 0.1, 0.01, 0.001, etc., respectively. (**c**) Estimated inactivation ratio for a characteristic fluence $F_i=5$ J/m^2^ (susceptibility $k= 0.2$ m^2^/J), and absorption coefficient $\alpha =0.5$ mm$^{-1}$ in spherical cap geometry (solid line) and cylindrical geometry (dotted line). The calculation has been done for three heights $h_{\mathrm{cyl}}=h_{\mathrm{cap}}=h=3, 6$ and 9 mm (red, green, and blue plots, respectively). The black thin solid line is for a non-absorbing sample ($\alpha =0$).

Experimentally, we have an easy access to the volume of the drop, $V_{\mathrm{cap}}$, and to the diameter of the circular bottom surface in contact with the substrate, 2*a*. Using these previous relations, it is possible to fully characterize the geometry of the spherical cap and calculate the radius, *r*, and central height, $h_{\mathrm{cap}}$. For this geometry, we can evaluate the number of surviving viruses by considering absorption when light travels through the drop in terms of the distance between these layers to the external interface exposed to the UV radiation. As far as we have assumed a collimated illumination, the successive layers are obtained by a parallel translation of the spherical surface of radius *r* that moves away from the air/liquid interface within the droplet (see Fig. 2b). The volume of the layer decreases when moving deeper within the droplet (see blue line in Fig. 3a) and is given by equation

11 $$\begin{aligned} \Delta V_{\mathrm{cap}} (z) = \pi (h_{\mathrm{cap}}-z) [ 2r-(h_{\mathrm{cap}}-z) ] \Delta z . \end{aligned}$$

This corresponds to the volume between two spherical caps of heights $h_{\mathrm{cap}}-z$ and $h_{\mathrm{cap}}-(z+\Delta z)$ where $z=0$ corresponds to the location of the apex of the droplet. If we assume a homogeneous virus distribution within the droplet, the number of viruses located within this $\Delta V_{\mathrm{cap}}$ volume is

12 $$\begin{aligned} \Delta N_0 (z) = N_0 \frac{3(h_{\mathrm{cap}}-z)[2r-(h_{\mathrm{cap}}-z)]}{h_{\mathrm{cap}}^2(3r-h_{\mathrm{cap}})} \Delta z . \end{aligned}$$

This dependence is plotted in Fig. 3a as a blue solid line: the contribution to the total number of viruses decreases from the outside of the droplet towards the core of the spherical cap. In case of a cylindrical geometry, the value of $\Delta N_0(z)$ is constant. By adding together all the contributions from the layers within the spherical cap, we can obtain the modeled survival ratio for this geometry as:

13 $$\begin{aligned} \eta _{\mathrm{cap}} (F) = \int _0^{h_{\mathrm{cap}}} \frac{3(h_{\mathrm{cap}}-z)[2r-(h_{\mathrm{cap}}-z)]}{h_{\mathrm{cap}}^2(3r-h_{\mathrm{cap}})} \exp \left[ - \frac{ F T}{F_i} \exp ( - \alpha z ) \right] dz. \end{aligned}$$

Equations (8) and (13) can be related to the integral-exponential function of the first kind^40,41^ to obtain an analytical solution. In this work, we made a computational evaluation of the functions by slicing the sample into a sufficiently large number of layers (approaching the case of $\Delta z \rightarrow 0$).

To further understand the interpretation of the models and the differences between both geometries, we have compared them by calculating some parameters of interest. In Fig. 3a, we display the fluence attenuation (in red) as we move from the outside of the sample towards its inner region. In Fig. 3b, we show the local survival ratio as a function of the location within the drop *z* (normalized to the characteristic distance $z_0=1/\alpha$); and the incident fluence, *F*, also normalized to the characteristic inactivation fluence $F_i$. The dashed line corresponds to a local survival rate of 0.37, and the solid lines labeled as -1, -2, etc. represent local survival rate of 0.1, 0.01 and so on (related with the logarithmic representation of $\eta$). This map shows that layers closer to the air/liquid interface ($z/z_0$ close to 0) are more exposed to local higher fluences, and show lower survival rate locally. However, as we move deeper into the sample (increasing $z/z_0$), the available energy is lower and the survival rate, $\eta$, increases. This explains why a thicker drop may show survival ratios above the expectations. It will also provide higher (and inaccurate) values for the inactivation characteristic fluence if absorption is ignored or miscalculated. This occurs when stirring is unavailable: the pathogens located at the bottom are shielded by upper layers, which absorb radiation and preclude their inactivation. The map in Fig. 3b is valid for both the cylindrical and the spherical cap models.

Furthermore, we explored how the models predict the inactivation at high fluences (or equivalent high extinction ratios).

 We first considered the case of different well depths for the cylindrical model, $h_{\mathrm{cyl}}$, and the equivalent heights for the spherical cap geometry, $h_{\mathrm{cap}}$. The values for this analysis are $h_{\mathrm{cap}}= h_{\mathrm{cyl}}=h=3, 6$ and 9 mm, a characteristic fluence $F_i=5$ J/m^2^ (susceptibility $k=0.2$ m^2^/J), and an absorption coefficient $\alpha =0.5$ mm$^{-1}$. In Fig. 3c, we see how the inactivation curves for the three heights rapidly diverge from the non-absorbing case (plotted as a black solid line), and differ between both models (the spherical cap is plotted with a solid line and the cylindrical case with a dotted line). In this figure, the cylindrical model has a larger survival ratio than the spherical cap model for a fixed characteristic fluence: the layers exposed to lower fluences weigh less (have less volume and less pathogens) in the spherical cap model compared to the cylindrical one. The calculated values for the global survival ratio, $\eta$, vary several orders of magnitude when changing the height of the sample for high fluences. To include absorption, Morowitz^27^ suggests to introduce correction factors related with the average fluence, but this only applies when stirring is possible. Neglecting absorption may lead to significant errors when predicting inactivation ratio at high fluences as we have seen from the calculations shown in Fig. 3c. Also, an inappropriate choice of the model’s geometry may generate considerable divergences in the analysis and may provide unreliable results.

Equations (8) and (13) incorporate the fluence distribution within the sample. The difference between them is related with the geometry of the model through the term before the outer exponential function. In the next section we compare both geometries (cylindrical and spherical cap) and models (absorbing and non absorbing) to check their robustness when fitting the measurements. We implemented the algorithm that fits the experimental data to the model in Matlab (The MathWorks Inc. Natick, Massachusetts, USA). We minimized a merit function, *Q*, defined as the sum of the squared differences—evaluating their logarithms first—between the measured values and those derived from the model,

14 $$\begin{aligned} Q= \sum _{j=1}^N w_j \left[ \log _{10} \eta _j - \log _{10} \eta _{\mathrm{model}} ) \right] ^2 , \end{aligned}$$

where $w_j$ is defined as a weight for each datum that is related to measurement uncertainty. This weight is defined as the inverse of the squared relative error associated to each measured point. This previous merit function is automatically evaluated to provide a fitted value of $F_i$. *Q* can be also interpreted as the summed square residuals, $\text{ SSE }$. This parameter is part of the definition of the *R*-square, $R^2$, that describes the goodness of the fit:

15 $$\begin{aligned} R^2= 1- \frac{\text{ SSE }}{\text{ SST }} ,\end{aligned}$$

where $\text{ SST }$, the sum of the squares about the mean, is evaluated as:

16 $$\begin{aligned} \text{ SST }= \sum _{j=1}^N w_j \left[ \log _{10} \eta _j - \overline{\log _{10} \eta _j} ) \right] ^2 ,\end{aligned}$$

being $\overline{\log _{10} \eta _j}$ the mean of the $\log _{10}$ of the measured data, $\eta _j$. Since $\text{ SST }$ only depends on the dataset, if *Q* decreases, then $R^2$ increases. Therefore, we can conclude that the fitting method applied in this contribution finds the curve that best correlates (higher $R^2$) with the given data.

## Results and discussion

### Preliminary results for the inactivation

**Figure 4:**
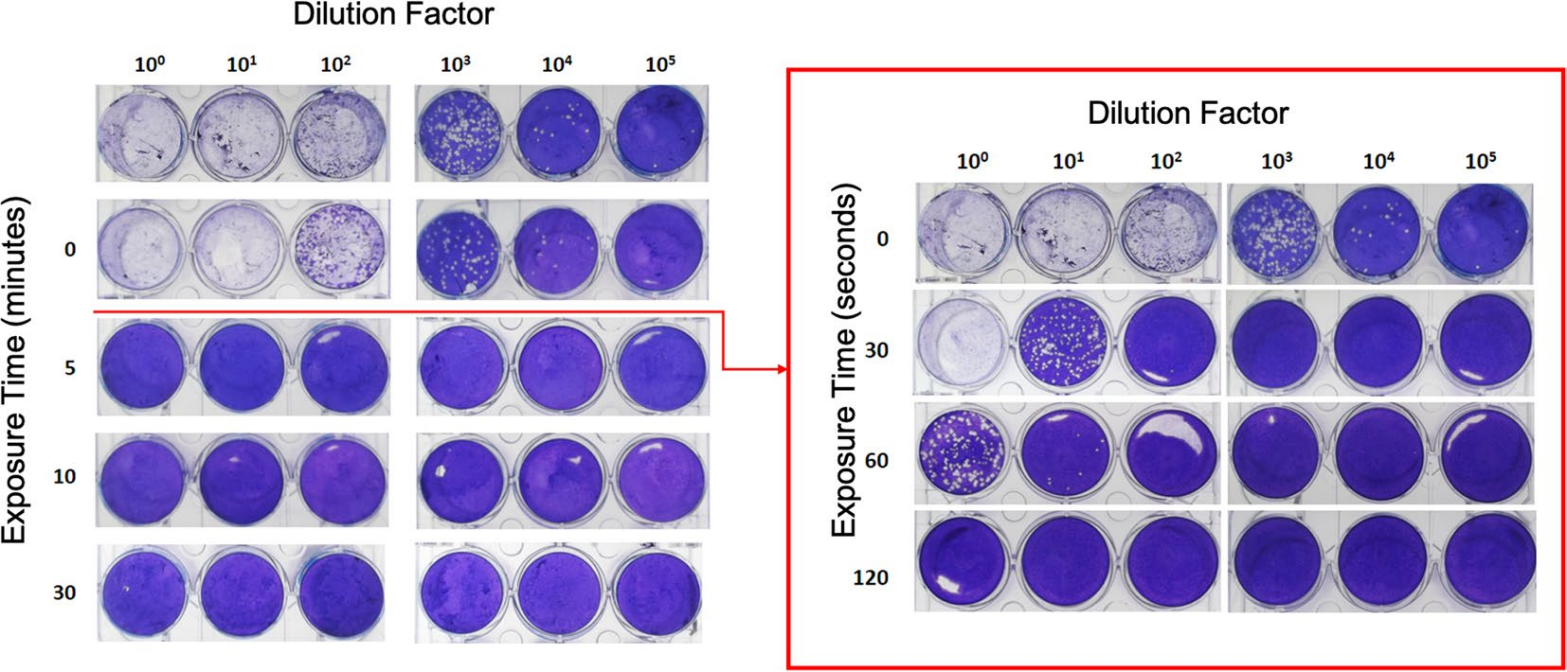
Representative image of cytophatic effect an viral plaque formation in samples exposed to UV-C light at the indicated times for an irradiance of $\sim 14$ W/m^2^ (see Irradiation chamber section). The red box on the right expands the exposure time between 0 and 2 min.

With a high value of irradiance ($\sim$14 W/m^2^), we first tested the inactivation potential of UV-C for virus-containing drops of 300 $\mu$L. These droplets were placed on borosilicate slides and exposed to UV-C irradiation for increasing amounts of time (0–5–10–30 min). After 5 min, the virus was completely inactivated to the limit of detection of the assay, which is $\le$ 1 PFU per mL (see Fig. 4). In a second validation experiment, the exposure time was decreased from 0 to 5 minutes. The virus was completely inactivated with an exposure between 60 to 120 seconds. This is a 400-fold decrease in infectious virus after 30 seconds of exposure. After three consecutive blinded passages in Vero E6 of the 120s already treated sample, no virus could be recovered. This indicated the full inactivation of SARS-CoV-2 through UV irradiation. After these preliminary results, our measurements were done with lower irradiance (and longer irradiation times) to retrieve the characteristic fluence of inactivation of the virus, $F_i$.

### Fitting to the models

Along the measurement process, we identified several sources of uncertainty that may be estimated as follows (an equivalent to 1 standard deviation, or a coverage factor $K=1$)^42^: (i) the estimation of the fluence $F=Et$ has a relative uncertainty of 5 % for the irradiance, *E*, and 10% for the period of exposure, *t*; (ii) the titer dilution and volume present a relative uncertainty $\sim$15%; (iii) the values of PFU range between 5 to 130 PFU, both determine $N_s$ and $N_0$. This results in a relative uncertainty that ranges between 10% and 60% for $\eta$. These sources of uncertainty add up in quadrature in relative terms and are represented in logarithmic scale. They generate an uncertainty of $\Delta \log _{10}\eta$ varying between $\pm 0.1$, and $\pm 0.3$, depending on the experiment and sample. These uncertainty values are included in the error bars in the figures and to calculate the weight for each measurement (see Eq. (14)).

**Figure 5:**
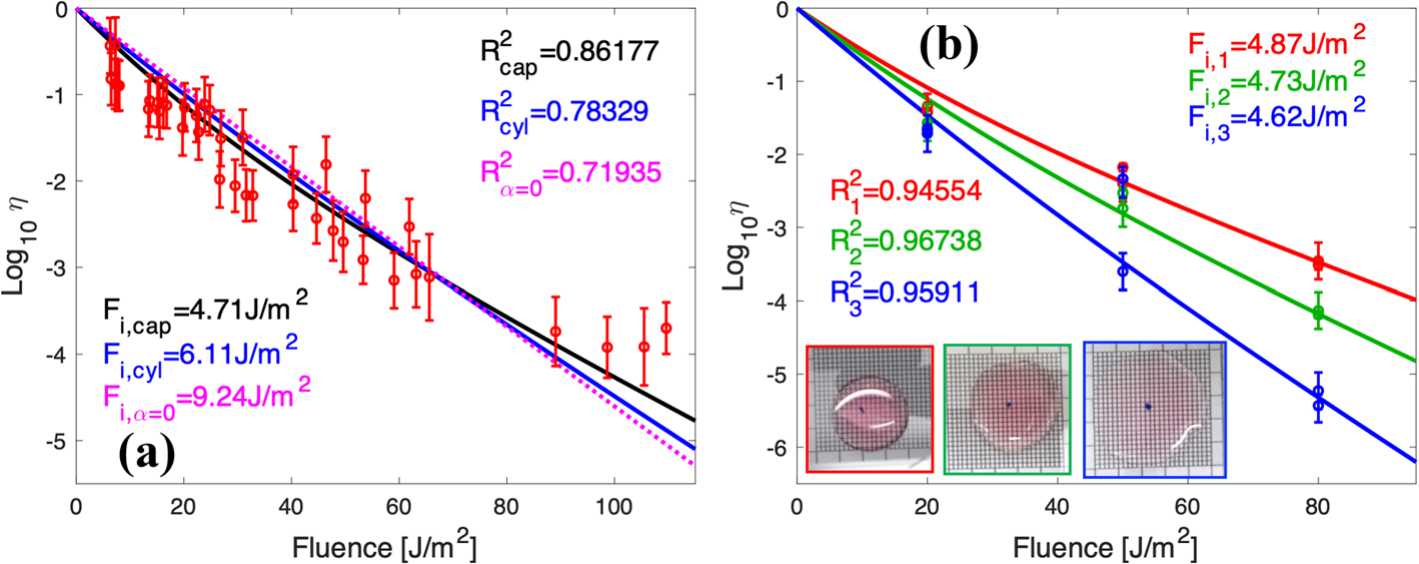
(**a**) Fitting of the data using the spherical cap model (in black) and the cylindrical model (in blue). The dashed magenta line corresponds with these two models for the case of a non-absorbing media ($\alpha =0$). The values of the characteristic fluence, $F_i$, are shown in the plot with the same color code. These fluences have been obtained changing the exposure time for an irradiance $\sim 0.7$ W/m^2^ (see Irradiation chamber section). (**b**) Fitting of three controlled experiments using drops of different heights with a fixed volume. Therefore, the height of the sample varies when changing the transversal size of the drop (see Table 1). The diameter of the drop increases as $2a_1=16.9$ mm (red), $2a_2=19.2$ mm (green), and $2a_3=24.2$ mm (blue). The value of the characteristic inactivation fluence is $\sim$ 4.7 J/m^2^. The error bars in plots (**a**) and (**b**) were obtained considering all uncertainty sources in the experiment. The inset shows three upper views of the drops framed with the same color code than the plots. The value of $R^2$ (see Eq. (15)) is also included within the plots to quantify the quality of the fitting. This fitting goodness parameter is larger in (**b**) due to the lower number of data points than in (**a**).

We applied the models described in Model of UV-C propagation through absorbing media section to a collection of data obtained from several measurement batches following the methodology presented in Virus sample preparation and UV irradiation section. Fig. 5.a shows the fitting of 40 measurements at several values of fluence for two different solutions of the virus. All data are obtained for droplets with a diameter $D=2a \simeq 16.9$ mm and a volume of $V_{\mathrm{cap}}=300 \mu$L. We show the experimental data in red. Our fitting with the spherical cap model provides a value of $F_{i,{\mathrm{cap}}}=4.71$ J/m^2^ (black solid line). In the case of the cylindrical model, we maintained the volume constant and recalculated the height to obtain a cylindrical shape with the observed diameter $D=2a \simeq 16.9$ mm. This calculation yields to a height lower than for the spherical cap model. Therefore, the cylinder is shallower than the spherical cap, and more liquid is exposed to higher fluences. To fit the experimental values, the characteristic fluence must be larger than in the spherical cap case. We found $F_{I,{\mathrm{cyl}}}=6.11$ J/m^2^ (blue solid line). As a final check, we also fitted the experimental results for the case of a non-absorbing media, $\alpha =0$. This considers that the sample is homogeneously exposed to the same fluence. As expected, the fitting provides a value of $F_{i,\alpha =0}=9.24$ J/m^2^ (magenta dotted line), which is roughly twice the value obtained for the spherical cap model.

**Table 1:** Calculated values of the inactivation characteristic fluence, $F_i$, when fitting the experimental values obtained for three drops, to the cylindrical, spherical cap, and non absorbing ($\alpha =0$) models. The volume for all the drops is the same $V=300 \mu$L.

2*a* [mm]	$h_{\mathrm{cap}}$ [mm]	$F_{i,{\mathrm{cap}}}$ [J/m^2^]	$h_{\mathrm{cyl}}$ [mm]	$F_{i,{\mathrm{cyl}}}$ [J/m^2^]	$F_{i,\alpha =0}$ [J/m^2^]
16.9	2.6	4.87	1.3	6.28	9.42
19.2	2.0	4.73	1.0	5.87	8.04
24.2	1.3	4.62	0.7	5.20	6.28

To understand how the geometry of the droplet may affect experimental results, we performed two batches of measurements for three spread drops of different diameters with a fixed volume, $V_{\mathrm{cap}}=300 \mu$L. The diameters of the droplets were $2a_1=16.9 \pm 0.2$ mm, $2a_2=19.2 \pm 0.3$ mm, and $2a_3=24.2 \pm 0.4$ mm. Even though the measurements are located along different fitted curves of the spherical cap model (Eq. (13)), all of them generate a value of $F_i \sim 4.7$ J/m^2^ (see Table 1 and Fig. 5b). This value is very similar to the one in the fitting shown in Fig. 5a. The wider droplet produces data with a lower survival ratio, $\eta$. This is expected for a shallower drop because light can easily penetrate into a larger volume. However, although the survival ratio at the highest fluence, $F=80$ J/m^2^, differs in almost two orders of magnitude between the wider and the narrower droplets, the fitted characteristic fluence, $F_{i, {\mathrm{cap}}}$, merely varies $\sim$3%. We fitted the same experimental data to the cylindrical geometry of our model (see Eq. (8)). We maintained the sample’s volume constant and, as a consequence, the cylinder’s height differs from the height of the spherical cap: $h_{\mathrm{cyl}} < h_{\mathrm{cap}}$. For the cylindrical geometry (see Table 1), our results for the characteristic fluence, $F_{i, {\mathrm{cyl}}}$, show a large variability (around 8%). However, since the cylindrical geometry is thinner than the spherical cap geometry, the fitted value of the characteristic fluence is higher. Moreover, when fitting this dataset to the non-absorbing case, the variation of the fitted characteristic fluence, $F_{i, \alpha =0}$ was $\sim$20% (even larger than the calculated $F_{i,{\mathrm{cyl}}}$). Our results show that the spherical cap model as the best model to extract a reliable value of $F_i$.

## Conclusions

In this paper, we revisited the analytic and experimental strategies of UV inactivation kinetics experiments by including an accurate description of the optical absorption of culture media, and the evaluation of the local fluence within the sample. Our analytical model is valid for two sample geometries: cylindrical and spherical cap. We measured the pathogen’s survival ratio for a wide range of fluence values and we have fitted them to three models: cylindrical geometry, spherical cap shape geometry, and non-absorbing culture medium. We found discrepancies in the characteristic fluence derived from the models, so we continued to further refine our analysis to explain realistically the observed phenomenon.

We found that the modeled geometry is key for a representative fit: it must reflect the experimental sample’s shape. To validate the geometry modeled, we analyzed three spread drops with equal volume and different transversal size but with the same spherical cap geometry. Each droplet showed a different survival rate: over an order of magnitude for the highest fluence. However, when we fitted them with the spherical cap model, we found that the characteristic fluence for the three cases was quite similar. Moreover, it was also very close to the one obtained for a larger number of drops having a constant transversal size. If these data are fitted with the cylindrical geometry model or the non-absorbing case, the characteristic fluence changes significantly among drops. As a result, we found that neglecting absorption and choosing a non-appropriate geometry can overestimate the required UV dose for a fixed inactivation level. Optical absorption is especially relevant in the interpretation of high inactivation ratios and/or doses. Our results suggest that the role of optical absorption alone may explain many of the large discrepancies found in other works, even comparing the same pathogen^8^. We have shown analytically and experimentally how the geometry of the model – how absorption occurs in a droplet with a spherical cap shape – improves the robustness in the determination of the characteristic fluence. This refined geometry better models the experimental results when compared to the simpler cylindrical model, specially when comparing drops with different geometrical parameters. Our analytic functions $\eta _{\mathrm{cyl}}$ and $\eta _{\mathrm{cap}}$ can aid to analyze experimental results according to the applicable geometry.

After improving the reliability of the chosen analytical model, we determined the characteristic inactivation fluence of the SARS-CoV-2 pathogen at $\lambda _{\mathrm{Hg}}= 254$ nm. This value is $F_i=4.7 \pm 0.1$ J/m^2^, indicating a high susceptibility to UV-C of $k=0.21 \pm 0.01$ m^2^/J. From the characteristic fluence, $F_i$, it is possible to calculate the values of the fluences for any extinction ratio (D50, D90, and D99). For example, we obtain $F_{\mathrm{D}90}=10.8 \pm 0.2$ J/m^2^. However, these fluences are only valid if $F_i$ corresponds to the value obtained for the most trusted geometry and when optical absorption is considered. The determination of the characteristic fluence is key when evaluating the capability of UV inactivation of the SARS-CoV-2. $F_i$ represents the effect of the radiation on the pathogen and removes the optical effects of the surrounding media and the geometrical arrangements, similar to exposing a bare virus directly to UV radiation. We believe that our results will help other researchers to further understand their results and to obtain reliable values of virus survival rate when exposed to radiation. Especially, in situations where the virus is immersed in media with specific optical properties, which may be the case of micro-droplets emitted by infected individuals who generate airborne pathogens. As a summary, our results contribute to the determination of the capabilities of UV disinfection strategies from experiments made in culture media. The models presented here can be applied to any wavelength and pathogen. Besides, the improved reliability of $F_i(\lambda )$ for the given pathogen (including SARS-CoV-2) should help to obtain the inactivation action spectrum of UV-C light.
